# The effect of the environment on symptom dimensions in the first episode of psychosis: a multilevel study

**DOI:** 10.1017/S0033291713003188

**Published:** 2014-01-20

**Authors:** F. J. Oher, A. Demjaha, D. Jackson, C. Morgan, P. Dazzan, K. Morgan, J. Boydell, G. A. Doody, R. M. Murray, R. P. Bentall, P. B. Jones, J. B. Kirkbride

**Affiliations:** 1 Department of Psychiatry, Herchel Smith Building for Brain and Mind Sciences, University of Cambridge, UK; 2 Faculty of Medicine, Lund University, Sweden; 3 NIHR Biomedical Research Centre, Psychosis Studies Department, Institute of Psychiatry, King's College London, UK; 4 MRC Biostatistics Unit, University of Cambridge, UK; 5 NIHR Biomedical Research Centre and Section of Social Psychiatry, Health Service and Population Research Department, Institute of Psychiatry, King's College London, UK; 6 Department of Psychology, University of Westminster, London, UK; 7 Division of Psychiatry, University of Nottingham, UK; 8 Institute of Psychology, Health and Society, University of Liverpool, UK; 9 Division of Psychiatry, University College London, UCL

**Keywords:** Epidemiology, neighbourhood, paranoia, positive symptoms, psychosis, symptom dimensions, symptomatology, social environment, urbanicity

## Abstract

**Background:**

The extent to which different symptom dimensions vary according to epidemiological factors associated with categorical definitions of first-episode psychosis (FEP) is unknown. We hypothesized that positive psychotic symptoms, including paranoid delusions and depressive symptoms, would be more prominent in more urban environments.

**Method:**

We collected clinical and epidemiological data on 469 people with FEP (ICD-10 F10–F33) in two centres of the Aetiology and Ethnicity in Schizophrenia and Other Psychoses (AESOP) study: Southeast London and Nottinghamshire. We used multilevel regression models to examine neighbourhood-level and between-centre differences in five symptom dimensions (reality distortion, negative symptoms, manic symptoms, depressive symptoms and disorganization) underpinning Schedules for Clinical Assessment in Neuropsychiatry (SCAN) Item Group Checklist (IGC) symptoms. Delusions of persecution and reference, along with other individual IGC symptoms, were inspected for area-level variation.

**Results:**

Reality distortion [estimated effect size (EES) 0.15, 95% confidence interval (CI) 0.06–0.24] and depressive symptoms (EES 0.21, 95% CI 0.07–0.34) were elevated in people with FEP living in more urban Southeast London but disorganized symptomatology was lower (EES –0.06, 95% CI –0.10 to –0.02), after controlling for confounders. Delusions of persecution were not associated with increased neighbourhood population density [adjusted odds ratio (aOR) 1.01, 95% CI 0.83–1.23], although an effect was observed for delusions of reference (aOR 1.41, 95% CI 1.12–1.77). Hallucinatory symptoms showed consistent elevation in more densely populated neighbourhoods (aOR 1.32, 95% CI 1.09–1.61).

**Conclusions:**

In people experiencing FEP, elevated levels of reality distortion and depressive symptoms were observed in more urban, densely populated neighbourhoods. No clear association was observed for paranoid delusions; hallucinations were consistently associated with increased population density. These results suggest that urban environments may affect the syndromal presentation of psychotic disorders.

## Introduction

Traditional epidemiological studies of first-episode psychosis (FEP), underpinned by categorical diagnostic classifications, have identified major risk factors for both non-affective and affective psychotic disorders. Risk factors for these sets of disorders are shared [migration and ethnicity (Cantor-Graae & Selten, 2005; Fearon *et al.* 2006) and childhood traumas (Laursen *et al.* 2007)] and unique [e.g. urban birth and upbringing (Mortensen *et al.* 1999), paternal age (Laursen *et al.* 2007), developmental delays (Jones *et al.* 1994) and impaired pre-morbid cognition in schizophrenia (Reichenberg *et al.* 2002)], suggesting that there may be both overlapping and distinct aetiological pathways to psychotic disorder.

In the past decade psychiatric research has questioned the diagnostic utility of traditional categorical classifications (Liddle, 1987; Demjaha *et al.* 2009; van Os *et al.* 2010), suggesting that dimensional conceptualizations of symptomatic presentation may also be useful in characterizing the true psychopathology underlying psychotic illness (van Os *et al.* 1996; Peralta & Cuesta, 2007; Braca *et al.* 2013; Russo *et al.* 2013). Some studies have investigated how individual-level risk factors for categorical diagnoses map onto different psychosis symptom dimensions (Stefanis *et al.* 2004; Allardyce *et al.* 2007; Demjaha *et al.* 2009) but only one study has examined this in relation to environmental factors (van Os *et al.* 2002). In that study only positive and negative symptoms were considered and multilevel modelling was not used.

In the current study we examined the extent to which several psychosis symptom dimensions in people with FEP exhibited variance according to measures of the social environment. We hypothesized that urban living would be most strongly associated with positive psychotic symptom dimensions, and within these dimensions, specifically with paranoia. Psychological models propose that paranoid symptoms exist on a continuum with healthy functioning (Freeman *et al.* 2005; van Os *et al.* 2009), co-opt healthy processes for detecting and avoiding social threats (Moutoussis *et al.* 2007) and arise from pre-existing feelings of vulnerability (Freeman *et al.* 2002) and low self-esteem (Bentall *et al.* 2001). There is evidence that positive symptoms in general (Schreier *et al.* 2009), and paranoia in particular (Janssen *et al.* 2003; Bentall *et al.* 2012), can develop following experiences of victimization, trauma and discrimination, and in the general population it seems plausible that these kinds of experiences would be more frequently encountered in high-density, urban environments. Consistent with this hypothesis, following exposure to urban environments, paranoid individuals exhibit increased levels of anxiety, negative beliefs about others and a tendency to ‘jump to conclusions’ on the basis of limited data (Ellett *et al.* 2008). We also hypothesized that depressive symptoms would be more common in people with FEP in more urban environments because mood disorders, including unipolar depression, are elevated in urban areas (Sundquist *et al.* 2004; Peen *et al.* 2010). We had no strong *a priori* rationale to consider that other symptom dimensions would vary according to urban gradients.

## Method

### Sample

All participants who presented to services with a suspected FEP over a 2-year period (1997–1999) in the Southeast London and Nottinghamshire centres of the Aetiology and Ethnicity in Schizophrenia and Other Psychoses (AESOP) study were eligible for inclusion (Kirkbride *et al.* 2006; Demjaha *et al.* 2009). Our Southeast London centre included the Borough of Lambeth and two-thirds of the Borough of Southwark. The Nottinghamshire catchment area included the City of Nottingham and the more suburban and rural parts of the surrounding region, including the Local Authority Districts of Broxtowe, Gedling and Rushcliffe, and the town of Hucknall. We did not include participants from a third centre (Bristol) because symptom data were not collected there. Participants had to (i) be 16–64 years old, (ii) live in the catchment areas at first contact and (iii) not have an organic basis to disorder (including possible neurological and metabolic disorders) or profound learning disability. Services bases were monitored weekly, and a leakage study minimized loss to ascertainment (Kirkbride *et al.* 2006). The Schedules for Clinical Assessment in Neuropsychiatry (SCAN; WHO, 1992) were administered to eligible participants as soon as possible after first contact. Participants were diagnosed using the SCAN and other available information (including case-note review and informant interview) by a clinical panel. Participants who met ICD-10 criteria for substance-induced psychosis (F10–F19), non-affective psychosis (F20–F29), bipolar affective psychosis (F30 and F31) or psychotic depression (F32 and F33) were included.

### Data collection

#### Symptom dimensions

A.D. rated 28 major signs and symptoms of psychosis on all participants according to the SCAN Item Group Checklist (IGC), using the SCAN and all available clinical data. IGC ratings were based on both frequency and severity, and were coded as absent, moderate or severe (rated 0, 1 and 2 respectively). Ambiguous ratings were resolved by consensus with P.D. Our group has previously reported the factor structure underlying these IGC symptom groups in the AESOP FEP sample, and their association with individual-level clinical and sociodemographic characteristics (Demjaha *et al.* 2009). In that study, a principal axis factor analysis with varimax rotation suggested that five theoretically informed and empirically driven symptom dimensions could represent symptomatology: reality distortion, negative symptoms, manic symptoms, depressive symptoms and disorganization.

In the current study we investigated the extent to which each of these symptom dimensions varied at the small-area neighbourhood level. We obtained the factor loading matrix of IGC items following principal axis factor analysis with varimax rotation (Supplementary Table 1, ST1) in Stata version 12 (Stata Corporation, USA), and calculated participant-level factor scores on each symptom dimension using Stata's predict post-estimation command following factor analysis. Because scores were highly positively skewed (data available on request), we applied zero-skew transformations to each symptom dimension to minimize violating the assumption that residuals in our final multilevel regression models were normally distributed.

#### IGC symptom clusters and items

To test the *a priori* hypothesis that paranoid symptoms were specifically elevated in more urban environments, we analysed each IGC symptom item as a separate outcome in multilevel, multivariable analyses. Two IGC items on the reality distortion dimension (see ST1) were of specific interest with regard to paranoia, delusions of persecution and delusions of reference. For the reality distortion dimension we also examined whether clinical symptom clusters varied at the neighbourhood level. For each participant, IGC symptom item scores were summed to produce three ordinal clusters within the reality distortion dimension: delusions (delusions of persecution, delusions of reference, delusions of control, bizarre delusions and interpretations, miscellaneous delusions; scores ranged from 0 to 12), hallucinations (non-affective auditory hallucinations, non-specific auditory hallucinations, non-specific visual hallucinations, altered perception; scores ranged from 0 to 8) and other symptom items associated with reality distortion (non-specific psychotic experiences, experiences of disordered form of thoughts, depersonalization and derealization; scores ranged from 0 to 6).

#### Individual-level sociodemographic and clinical variables

We recorded sex, age, ethnicity, highest socio-economic position and marital status (single, married, divorced/separated, widowed) at first contact for all participants using the Medical Research Council Sociodemographic Schedule (Fearon *et al.* 2006). Ethnicity was collapsed into seven categories: white British, white non-British, black Caribbean, black African, Indian subcontinent (Indian, Pakistani, Bangladeshi), mixed white and black Caribbean, and all other ethnicities. Highest ever socio-economic position was based on occupation, coded using the National Statistics Socio-economic Classification (NS-SEC; ONS, 2005) with six categories: professional, self-employed and intermediate occupations, supervisory roles, semi-routine occupations, routine occupations, long-term employed. We included a broad categorical ICD-10 diagnosis variable as a potential confounder, as described earlier. In addition, we recorded mode of onset [acute (<1 month) *versus* insidious (>1 month)], lifetime poly-drug use prior to first contact (no use, single drug use, poly-drug use) and parental history of psychosis from the Personal and Psychiatric History Schedule, Schedule for Drug Use Assessment, Family Interview for Genetic Studies and all other data sources in the AESOP study.

#### Area-level variables

We included a centre-level variable to inspect whether symptom dimensions differed between our more urban [Southeast London, 2001: 95 people per hectare (pph)] and less urban (Nottinghamshire, 2001: 30 pph) settings. To determine whether symptom dimensions varied across smaller neighbourhoods, we geocoded participants to Office for National Statistics 2001 statistical wards (henceforth, the ‘neighbourhood’ or ‘neighbourhood level’) in which they resided at first contact with services for FEP (*n* = 88 neighbourhoods; mean population: 9650). Participants of no fixed abode or who could not otherwise be geocoded were excluded. We estimated several neighbourhood-level socio-environmental exposures using data collected as close as possible to first contact, including: population density (in pph, 2001 census) (Kirkbride *et al.* 2007, 2012), own-group ethnic density (as a proportion of total neighbourhood population, 2001 census) (Kirkbride *et al.* 2007, 2012), deprivation [Index of Multiple Deprivation (IMD) scores, 2004 (Noble *et al.* 2004); calculated at lower super output area level and re-estimated at neighbourhood level based on a population-weighted mean (Kirkbride *et al.* 2007)], inequality [disparity in IMD scores across each neighbourhood at lower super output area level (Kirkbride *et al.* 2012); estimated using the Gini coefficient, where 0 indicated maximum equality (i.e. all lower super output areas in a neighbourhood were equally deprived) and 100 indicated maximum inequality] and 2002 local election voter turnout (percentage turnout in each neighbourhood) as a proxy for social capital (Kirkbride *et al.* 2007). All neighbourhood variables were *z* standardized with a mean of zero and standard deviation (s.d.) of one.

### Statistical analyses

#### Multilevel models

We used multilevel linear regression to inspect neighbourhood-level and centre-level differences in transformed symptom dimensions. For ordinal outcomes (symptom clusters and IGC items) we used multilevel ordinal regression. For some individual IGC items there was insufficient variation to fit ordinal regression models, so we collapsed the outcome into a binary variable (absent *versus* moderate/severe) and used multilevel logistic regression. All models were random intercept models that included a random effect to allow baseline symptomatology to vary between neighbourhoods, but assumed individual-level exposures had the same (fixed) effect across all neighbourhoods.

#### Modelling strategy

Treating each transformed symptom dimension separately, we first ran a null multilevel model to estimate the proportion of neighbourhood-level variance in the symptom dimension. We then added age, sex and ethnicity as *a priori* confounders to a model with our centre-level variable, which was retained as a fixed effect in further model building if it significantly improved model fit. Using a forward-fitting approach, we then added broad diagnostic category and other symptom dimensions to the model to see if they confounded our findings. Finally, we tested whether any neighbourhood-level socio-environmental variables improved the model fit. We reported results from the most parsimonious model for each symptom dimension. An analogous approach was adopted for ordinal IGC symptom clusters and items. Because of a substantial degree of missing data on five confounders (mode of onset, poly-drug use, parental history of psychosis, highest socio-economic position, marital status; see Table 1), we inspected their confounding effect on our results using sensitivity analyses. For each symptom dimension, we first included these five variables in the final model with missing values coded to their minimums (acute onset, no drug use, no family history, professional occupation, married) and reported the change in any neighbourhood-level effect size. We then repeated this procedure having coded missing data to their maximum values (insidious onset, poly-drug use, positive family history, long-term unemployed, single).

**Table 1.** Participant social and clinical characteristics

Sample characteristics at referral	Total, *N* (%)	Southeast London, *N* (%)	Nottinghamshire, *N* (%)	Test statistics^a^
Total cases	469 (100.0)	309 (65.9)	160 (34.1)	
Median age, years (IQR)	29 (22–36)	29 (23–36)	28 (21–37)	*z* = 0.94, *p* = 0.34
Sex				
Men	266 (56.7)	169 (54.7)	97 (60.6)	
Women	203 (43.3)	140 (45.3)	63 (39.4)	*χ*^2^ = 1.5, *p* = 0.22
Ethnicity^b^				
White British	196 (41.8)	72 (23.3)	124 (77.5)	
White, other ethnicities	34 (7.3)	29 (9.4)	5 (3.1)	
Black Caribbean and black African	126 (26.9)	176 (57.0)	12 (7.5)	
Indian subcontinent	14 (3.0)	4 (1.3)	10 (6.3)	
Mixed white and black Caribbean	10 (2.1)	5 (1.6)	5 (3.1)	
Other ethnicities	27 (5.8)	23 (7.4)	4 (2.5)	Fisher's *p* < 0.01
Highest socio-economic position				
Professional and managerial	44 (11.3)	24 (9.6)	20 (14.5)	
Intermediate and self-employed	89 (22.9)	69 (27.5)	20 (14.5)	
Supervisory occupations	29 (7.5)	14 (5.6)	15 (10.9)	
Semi-routine occupations	112 (28.8)	68 (27.1)	44 (31.9)	
Routine occupations	92 (23.7)	57 (22.7)	35 (25.4)	
Long-term unemployed	23 (5.9)	19 (7.6)	4 (2.9)	Fisher's *p* < 0.01
Missing^c^	80 [17.1]	58 [18.8]	22 [13.8]	
Marital status^b^				
Single	332 (74.4)	220 (75.6)	112 (72.3)	
Married	67 (15.0)	39 (13.4)	28 (18.1)	
Divorced or separated	43 (9.6)	n.r.	n.r.	
Widowed	4 (0.9)	n.r.	n.r.	Fisher's *p* = 0.47
Missing^c^	23 [4.9]	18 [5.8]	5 [3.1]	
Diagnosis^b^				
Non-affective psychosis	320 (68.2)	229 (74.1)	91 (56.9)	
Affective psychosis	128 (27.3)	n.r.	n.r.	
Drug-induced psychosis	21 (4.5)	n.r.	n.r.	Fisher's *p* < 0.01
Mode of onset				
Acute	211 (50.6)	141 (54.1)	70 (49.1)	
Insidious	222 (49.4)	147 (48.6)	75 (50.9)	*χ*^2^ = 1.5, *p* = 0.70
Missing^c^	36 [7.7]	21 [6.8]	15 [9.4]	
Lifetime poly-drug use				
No use	142 (40.2)	94 (41.2)	48 (38.4)	
Single drug use	100 (28.3)	75 (32.9)	25 (25.0)	
Poly-drug use	111 (31.4)	59 (25.9)	52 (41.6)	*χ*^2^ = 11.2, *p* < 0.01
Missing^c^	116 [24.7]	81 [26.2]	35 [21.9]	
Parental history of psychosis				
No	258 (86.0)	142 (85.0)	116 (87.2)	
Yes	42 (14.0)	25 (15.0)	17 (12.8)	*χ*^2^ = 0.3, *p* = 0.59
Missing^c^	169 [36.0]	142 [46.0]	27 [16.9]	

n.r., Not reported. To preserve participant anonymity some cells (where *n* < 3) are not reported by centre.

Values given as number (percentage) or median (interquartile range; IQR).

a *χ*^2^ test except for Fisher's exact test where stated when small cell values (<5) encountered, or for median comparison of age by centre (Wilcoxon rank sum test). Tests compare strata of non-missing cells only.

b For the ethnicity variable, black Caribbean and black African groups have been collapsed for presentation purposes.

c Total number of participants with missing data, expressed as a percentage of overall sample (*n* = 469) in square brackets.

#### Model fit and reporting

Model fit was assessed with Akaike's Information Criterion (AIC), with lower scores indicating a better fit. Estimated effect sizes (EES) and their 95% confidence intervals (CIs) refer to the change in the transformed symptom dimension given a unit change in each covariate. For ordinal or binary IGC outcomes, we report adjusted odds ratios (aORs) and 95% CIs associated with a unit change in a given covariate.

### Ethical standards

All procedures contributing to this work complied with the ethical standards of the relevant national and institutional committees on human experimentation and with the Declaration of Helsinki of 1975, as revised in 2008.

## Results

### Sample

Of the 535 participants who presented to services with FEP in the two centres, we had complete IGC data on 484 (90.5%). Five symptom dimensions have previously been reported to underlie IGC symptoms in this sample (Demjaha *et al.* 2009) (ST1). We excluded a further 15 participants because (i) they lived outside the catchments at first referral (*n* = 3), (ii) they had no fixed abode (*n* = 11), or (iii) their address at first presentation could not otherwise be geocoded (*n* = 1). A larger proportion of the Nottinghamshire sample was excluded [Nottinghamshire: 22.0% (*n* = 45) *versus* Southeast London: 6.4% (*n* = 21), *χ*^2^ = 28.4, 1 degree of freedom (df), *p* < 0.001]. Excluded participants did not differ on any other individual-level clinical or sociodemographic variables (data available on request).

The final sample for analysis was 469, of whom 56.7% were men (Table 1). The proportion of participants from an ethnic minority group (76.7% *v*. 22.5%, Fisher's exact *p* < 0.01) or receiving a diagnosis of non-affective psychosis (74.1% *v*. 56.9%, Fisher's exact *p* < 0.01) was greater in Southeast London than in Nottinghamshire; lifetime poly-drug use was more common in Nottinghamshire (41.6% *v*. 25.9%, *χ*^2^ = 11.2, 1 df, *p* < 0.01). A smaller proportion of participants in Southeast London compared with Nottinghamshire had attained a professional occupation at first referral but were more likely to be self-employed, in intermediary occupations or long-term employed; a larger proportion of the Nottinghamshire sample were in professional, semi-routine and routine occupations (Fisher's exact *p* < 0.01). No other statistically significant differences were observed between centres on any sociodemographic or clinical variables. Neighbourhoods in our Southeast London centre had higher median levels of population density, multiple deprivation and black and minority ethnic density, and had lower median levels of inequality and voter turnout at local elections compared with neighbourhoods in our Nottinghamshire centre (Table 2).

**Table 2.** Neighbourhood ward-level characteristics in the Southeast London and Nottinghamshire AESOP catchment areas

Neighbourhood characteristics	Total median (IQR)	Southeast London median (IQR)	Nottinghamshire median (IQR)	Wilcoxon rank sum *z* score; *p* value
Population density (pph)	42.7 (24.7–84.6)	94.8 (81.6–119.0)	30.2 (12.1–42.7)	*z* = 7.3; *p* < 0.01
IMD score	28.6 (15.5–38.1)	34.1 (28.6–38.4)	17.8 (10.2–33.1)	*z* = 3.6; *p* < 0.01
IMD Inequality (%)	14.0 (9.4–19.0)	11.0 (8.6–14.8)	15.3 (11.1–21.5)	*z* = –3.1; *p* < 0.01
Local election voter turnout (%)	30.2 (24.6–37.2)	24.6 (22.9–29.9)	34.5 (28.7–43.2)	*z* = –4.9; *p* < 0.01
Own-group ethnic density (%)				
White British^a^	84.1 (54.3–94.1)	51.3 (43.4–54.8)	91.6 (85.6–95.7)	*z* = –7.6; *p* < 0.01

AESOP, Aetiology and Ethnicity in Schizophrenia and Other Psychoses; IQR, interquartile range; pph, people per hectare; IMD, Index of Multiple Deprivation.

a For clarity of presentation, own-group ethnic density is summarized for the white British group only. In multilevel models neighbourhood own-group ethnic density is estimated for each ethnic group (*n* = 7).

### Multilevel modelling of psychotic symptom dimensions

Null multilevel models suggested that approximately 4.9% of variance in reality distortion (*χ*^2^ *p* = 0.03) and 3.1% of variance in disorganization (*χ*^2^ *p* = 0.05) could be attributed to the neighbourhood level (Supplementary Table ST2). For both symptom dimensions, univariate associations were observed with several neighbourhood-level exposures, including evidence that reality distortion was elevated in our more urban centre, Southeast London, but levels of disorganization were lower (ST2). Despite the absence of any apparent small-area neighbourhood variation in depressive symptoms, a univariate model suggested that greater symptomatology was present in the Southeast London sample. Univariate associations suggested reality distortion was also elevated in some ethnic minority groups, but otherwise there was little evidence that any symptom dimension varied by individual-level exposures.

**Fig. 1.**
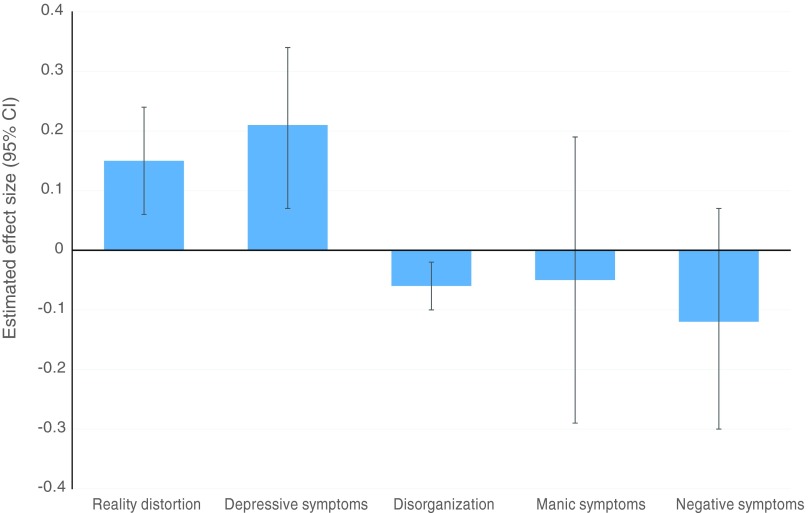
Estimated effect size (EES) of change in transformed symptom dimension scores for people with first episode psychosis (FEP) in the more urban, Southeast London centre of the Aetiology and Ethnicity in Schizophrenia and Other Psychoses (AESOP) study [mean population density: 95 people per hectare (pph)], compared with their counterparts in the less urban Nottinghamshire centre (mean population density: 30 pph). Values of EES are adjusted for age, sex, ethnicity, broad diagnosis and any other statistically significant symptom dimensions associated with the outcome variable (see Table 3). Positive values for EES (i.e. above the baseline, Nottinghamshire sample) indicate raised symptomatology in Southeast London whereas negative values indicate reduced symptomatology in Southeast London. Statistically non-significant differences between the two centres in symptomatology are indicated by 95% confidence intervals (CIs) that overlap zero.

Centre-level differences in reality distortion, depressive symptoms and disorganization remained evident after adjustment for age, sex, ethnicity, broad diagnosis and other symptom dimensions included in our final multivariate models (Fig. 1 and Table 3). Both reality distortion (EES 0.15, 95% CI 0.06–0.24) and depressive symptoms (EES 0.21, 95% CI 0.07–0.34) were elevated in Southeast London compared with Nottinghamshire, whereas levels of disorganization were lower (EES –0.06, 95% CI –0.10 to –0.02). These differences persisted after additional adjustment for mode of onset, poly-drug use, socioeconomic position, marital status and parental history of psychosis in sensitivity analyses (Table 4). Manic and negative symptom dimensions did not vary significantly between centres after full multivariate adjustment (Fig. 1). No specific neighbourhood-level exposure improved model fit (data available on request).

**Table 3.** Final multilevel models for symptom dimensions^a^ where significant area-level effects were observed

	Reality distortion EES (95% CI)	Depressive symptoms EES (95% CI)	Disorganization EES (95% CI)
Area-level fixed effects			
Southeast London *v*. Nottinghamshire	0.15 (0.06 to 0.24)	0.21 (0.07 to 0.34)	–0.06 (–0.10 to –0.02)
Individual-level fixed effects			
Ethnicity			
White British	1	1	1
White, other ethnicities	0.12 (–0.03 to 0.26)	–0.03 (–0.24 to 0.18)	0.009 (–0.05 to 0.07)
Black Caribbean	0.03 (–0.07 to 0.13)	–0.19 (–0.34 to –0.05)^b^	0.0002 (–0.04 to 0.04)
Black African	0.10 (–0.02 to 0.23)	–0.16 (–0.33 to 0.02)	–0.02 (–0.07 to 0.04)
Indian subcontinent	–0.01 (–0.22 to 0.21)	–0.14 (–0.45 to 0.16)	0.006 (–0.07 to 0.09)
Mixed, white and black Caribbean	–0.14 (–0.39 to 0.10)	–0.04 (–0.40 to 0.31)	0.04 (–0.05 to 0.14)
Other ethnicities	0.05 (–0.12 to 0.21)	–0.09 (–0.35 to 0.12)	0.005 (–0.06 to 0.07)
Diagnostic category			
Non-affective psychotic disorder	1	1	1
Affective psychotic disorder	–	–	–0.06 (–0.10 to –0.03)^b^
Bipolar psychotic disorder	–0.45 (–0.58 to –0.33)^b^	–0.12 (–0.31 to 0.07)	–
Depressive psychotic disorder	–0.23 (–0.33 to –0.12)^b^	0.86 (0.71 to 1.02)^b^	–
Substance-induced psychotic disorder	–0.12 (–0.30 to 0.06)	–0.20 (–0.46 to 0.06)	–0.05 (–0.12 to 0.02)
Symptom dimensions			
Manic symptoms	0.10 (0.07 to 0.13)^b^	0.11 (0.06 to 0.16)^b^	0.02 (0.006 to 0.03)^b^
Negative symptoms	–	–0.16 (–0.22 to –0.09)^b^	–
Area-level random effect: ICC (*χ*^2^ *p* value)	0.00% (*p* = 1.00)	0.18% (*p* = 0.47)	0.69% (*p* = 0.35)
AIC	471.06	809.87	–433.40

EES, Estimated effect size; CI, confidence interval; ICC, intraclass correlation coefficient; AIC, Akaike's Information Criterion.

a Effect sizes adjusted for age, sex and other variables in the final model, as presented. Effect sizes for age and sex not shown as no statistically significant associations with symptom dimensions were observed in the final models. All values rounded to two decimal places or one significant digit (where > –0.01 and < 0.01).

b Significant at *p* < 0.05.

**Table 4.** Sensitivity analysis to examine confounding effect of individual covariates containing missing data on area-level effects from final multivariate models: Southeast London versus Nottingham

	Reality distortion EES (95% CI)	Depressive symptoms EES (95% CI)	Disorganization EES (95% CI)
Sensitivity adjustment 1^a^	0.16 (0.06 to 0.25)^b^	0.21 (0.07 to 0.35)^b^	–0.06 (–0.09 to –0.02)^b^
Sensitivity adjustment 2^a^	0.16 (0.07 to 0.26)^b^	0.25 (0.11 to 0.39)^b^	–0.06 (–0.10 to –0.02)^b^

EES, Estimated effect size; CI, confidence interval.

a Final models from Table 3 with additional control for mode of onset, parental history of psychosis, lifetime poly-drug use, socio-economic position and marital status. Sensitivity adjustment 1 coded all people with missing data on these covariates to the baseline group [no evidence of lifetime drug use, acute first-episode psychosis (FEP) onset, no parental history, professional occupation, married]. Sensitivity adjustment 2 coded all people with missing data on these covariates to the highest exposure category (poly-drug use, insidious onset, positive parental history of psychosis, long-term unemployed, single).

b *p* < 0.05.

### Multilevel modelling of IGC symptom clusters and items

For IGC symptom clusters and items, population density provided a better fit to our models than the ‘centre’ variable (data available on request). Within the reality distortion dimensions, items related to hallucinations were most strongly and consistently associated with increased symptomatology among people with FEP living in more densely populated neighbourhoods (aOR associated with a 1 s.d. increase in population density: 1.32, 95% CI 1.10–1.61; Table 5). By contrast, we did not observed any consistent evidence that delusions (aOR 1.18, 95% CI 0.98–1.41), including delusions of persecution (aOR 1.01, 95% CI 0.83–1.23), were associated with population density, with the exception of delusions of reference (aOR 1.41, 95% CI 1.12–1.77). Two of the three other items associated with reality distortion (non-specific psychotic experiences, disordered form of thoughts) also showed positive associations with increased population density after adjustment for confounders (see Table 5).

**Table 5.** Association between population density and selected IGC symptom clusters (reality distortion, depressive symptoms and disorganization) and IGC symptom items

	aOR (95% CI)^a^	Multilevel regression model type^b^
Reality distortion		
Delusions	1.18 (0.98–1.41)	Ordinal logistic
Delusions of reference	**1.41 (1.12–1.77)** ^c^	Ordinal logistic
Delusions of persecution	1.01 (0.83–1.23)	Ordinal logistic
Delusions of control	1.18 (0.87–1.60)	Logistic
Bizarre delusions and interpretations	0.98 (0.78–1.23)	Ordinal logistic
Miscellaneous delusions	1.26 (0.99–1.60)	Logistic
Hallucinations	**1.32 (1.09–1.61)**	Ordinal logistic
Non-specific auditory hallucinations	**1.25 (1.001–1.55)**	Ordinal logistic
Non-specific visual hallucinations	1.39 (0.97–2.00)	Logistic
Non-affective auditory hallucinations	**1.33 (1.04–1.71)**	Ordinal logistic
Altered perception	**1.65 (1.15–2.36)** ^c^	Logistic
Other symptom items associated with reality distortion^e^	**1.39 (1.10–1.77)** ^d^	Ordinal logistic
Non-specific psychotic experiences	**1.56 (1.09–2.25)**	Logistic
Experiences of disordered form of thoughts	**1.39 (1.04–1.87)** ^d^	Ordinal logistic
Depersonalization and derealization	1.13 (0.80–1.60)	Logistic
Depressive symptoms		
Depressive delusions and hallucinations	**1.58 (1.00–2.48)** ^c^ ^,^ ^d^	Logistic
Depressed mood	**1.40 (1.11–1.76)**	Ordinal logistic
Special features of depressed mood	1.17 (0.89–1.52)	Logistic
Disorganization		
Emotional turmoil	**0.74 (0.60–0.92)**	Ordinal logistic
Incoherent speech	0.80 (0.59–1.08)	Ordinal logistic

IGC, Item Group Checklist; aOR, adjusted odds ratio; CI, confidence interval.

a Adjusted OR for 1 standard deviation increase in population density. OR adjusted for age, sex, ethnicity and broad diagnosis. Values in bold denote statistical significance at *p* < 0.05.

b Default modelling was multilevel ordinal logistic regression. For some outcomes there was insufficient variation in the ordinal outcome variable to permit ordinal logistic regression models. For these analyses a binary outcome variable (absent *versus* moderate/severe symptomatology) was used instead and multilevel logistic modelling performed.

c Additional adjustment for local election voter turnout, which significantly improved model fit.

d Additional adjustment for multiple deprivation, which significantly improved model fit.

e Other IGC symptom items that loaded substantively onto the reality distortion dimension (see Supplementary Table ST1).

For depressive symptoms, two of three IGC items on this dimension were also significantly associated with increased population density: depressive delusions and hallucinations (aOR 1.58, 95% CI 1.00–2.48) and depressed mood (aOR 1.40, 95% CI 1.11–1.76) (Table 5). Increased population density was associated with a lower odds of both IGC items on the disorganization dimension, although this only achieved statistical significance for ‘emotional turmoil’ (aOR 0.74, 95% CI 0.60–0.92).

## Discussion

### Principal findings

This is the first study to have investigated possible variation in symptom dimensions according to environmental factors in a FEP sample. We observed greater levels of reality distortion and depressive symptoms, and lower levels of disorganization in our most urban setting, after adjustment for several confounders. Manic and negative symptomatology showed little area-level variation. Delusions of persecution were not elevated among people experiencing FEP in more densely populated neighbourhoods in our sample, although one other symptom relevant to paranoid thinking, delusions of reference, did show such a relationship. Within the dimension of reality distortion, we observed that increased population density was most consistently associated with changes in hallucinatory symptomatology among people with FEP in our sample.

### Strengths and weaknesses

Symptom dimensions investigated in our study were based on theoretical and empirical evidence regarding underlying dimensional structure in FEP. Within the reality distortion dimension, we grouped IGC symptom clusters according to clinical knowledge; this is unlikely to have substantially biased our results. Greater endorsement of all hallucinatory IGC symptoms was associated with increased neighbourhood population density; this met conventional statistical significance on three of four items, with a strong trend in this direction for the fourth (non-specific visual hallucinations). We acknowledge that the cluster ‘other symptom items associated with reality distortion’ was heterogeneous. Clinically, it may be argued that symptoms of ‘depersonalization and derealization’ did not strictly constitute a ‘positive’ psychotic symptom, and this may have been borne out by the data because both ‘disordered form of thoughts’ and ‘non-specific psychotic experiences’ were significantly associated with population density, but this item was not. Given the novelty of our findings as a whole, however, and the diminished content validity inherent for any single IGC item, we suggest caution in the interpretation of results at the IGC item level.

Diagnostic data were collected by trained raters in each study centre using the SCAN, with high inter-rater reliability (between 0.6 and 0.8 for specific diagnoses, and 1.0 for psychotic disorder; Kirkbride *et al.* 2006). A single, trained rater conducted all SCAN IGC ratings to minimize differential ratings being applied between our centres. A greater proportion of participants missing IGC data (9.5% of the original sample) were from Nottinghamshire. If they were missing not at random (MNAR), this could have led to either under- or overestimation of symptom dimensions in this centre, affecting our results. Although we could not assess this directly, we consider that any such effect would have been small, given that excluded participants did not differ from the remainder of the sample on any clinical or social characteristics. Our FEP sample was not medication naïve at IGC assessment but, using available data (*n* = 173), we found no evidence to suggest that the proportion of antipsychotic-naïve participants at baseline differed statistically significantly between centres [Southeast London *n* = 18/77 (23.4%) *versus* Nottinghamshire *n* = 31/96 (32.3%); *χ*^2^ = 1.67, *p* = 0.20] (Pariante *et al.* 2005; Donoghue *et al.* 2012).

Our choice of confounders was guided by theoretical knowledge and the availability of data in the AESOP study. We did not control for duration of untreated psychosis (DUP) because our earlier work suggested that this did not vary at the neighbourhood level (Kirkbride *et al.* 2010). Categorical diagnoses met statistical significance for association with several symptom dimensions in our final models (Table 3), but did not substantially confound our main findings. Consistent with previous research (Allardyce *et al.* 2007; Demjaha *et al.* 2009; Braca *et al.* 2013) and theory (Peralta & Cuesta, 2007; van Os, 2009), these data suggest that both dimensional and categorical conceptualizations of psychosis may aid aetiological research. From a subgroup analysis, statistically significant area-level differences for depressive symptoms, reality distortion and disorganization were present in both the white British and black and minority ethnic groups (Supplementary Table ST3), making residual confounding by ethnicity an unlikely explanation for these area-level differences. Residual confounding may have been present with regard to drug misuse, for which we did not have data on frequency or dosage. Given the cross-sectional study design, we were unable to determine whether drug misuse may have mediated rather than confounded associations between urbanicity and symptom dimensions. More urban environments, for example, may increase exposure to substance misuse, increasing the risk of some psychotic symptoms. In the UK, available data suggest a small increase in declared illicit substance misuse in urban compared with rural areas (Smith & Flatley, 2011), although this may be confounded by several factors, including age and socio-economic position. Longitudinal studies are required to shed light on any mediating role for substance abuse between urban living and psychotic symptomatology.

Neighbourhood measures were collected as close as possible to the case ascertainment period. Population density was estimated from the 2001 census. Although this measure was estimated from data collected shortly after our case ascertainment period (2–4 years), we have no reason to believe that population density would have substantially altered across neighbourhoods during this time. It is possible that people with greater symptomatology in terms of reality distortion and depressive symptoms could have drifted into more densely populated environments as a result of social drift during the prodrome, but if this were true we would have expected to also observe greater levels of other symptom dimensions (negative, disorganized) in more urban environments; we did not.

### Meaning of findings

The novelty of our findings, in combination with the limitations outlined, mean that we encourage attempts to replicate our observations in other FEP samples. In this section, we place our findings in context with existing findings to suggest further directions that may be useful for future enquiry.

Our data support the notion that environmental factors act most strongly on positive psychotic symptoms related to reality distortion. Consistent with this possibility, further inspection of the three IGC items that loaded positively on the depressive symptoms dimension indicates that ‘depressive delusions and hallucinations’ (aOR 1.58, 95% CI 1.00–2.48) showed the largest effect size associated with population density (Table 5). These findings accord with current theoretical models of psychosis onset, which suggest that positive psychotic symptoms may be the result of salience dysregulation following exposure to deleterious genetic or social factors (Kapur, 2003). We suggest that densely populated environments may contribute to certain positive psychotic phenomena in two non-mutually exclusive ways.

First, growing up and living in a more densely populated, urban environment may simply increase exposure to environmental stimuli, including stimuli perceived as socially challenging, providing accumulated opportunities for dysregulation in salience and perception, perhaps through stress-related pathways (Lederbogen *et al.* 2011). Chronic exposure to the daily stresses of city life may, through a process such as neurochemical sensitization (Howes *et al.* 2004), render the brain liable to exaggerated dysfunction if additional environmental insults ensue (Pruessner *et al.* 2004). At the behavioural level, daily life stresses result in more psychotic-like experiences in people diagnosed with psychotic disorder and their first-degree relatives than controls (Myin-Germeys *et al.* 2005); this may be exaggerated in people previously exposed to major life events (Lardinois *et al.* 2011).

Second, the ‘status syndrome’ may be relevant to increased positive symptomatology seen for people with FEP in urban areas in this study. This hypothesis posits that ‘how much control you have over your life – and the opportunity you have for full social engagement and participation are crucial for health, well-being, and longevity’ (Marmot, 2004, p. 2). Cities may expose people to several sociocultural structures, organized, for example, around social, cultural, ethnic, political or economic modalities. The competing demands and complex interactions between these societal structures in an urban environment may impede an individual's control over their immediate social environment, acting as a source of social stress or marginalization among people who are (or who perceive themselves to be) excluded from full participation and representation in their community. By contrast, more rural communities, which are typically organized into more monocultural societies with smaller social hierarchies, may allow individuals greater control over their immediate environment.

The wider empirical data in mental health are consistent with this latter possibility. Our own research suggests that urban environments marked by more social fragmentation (Allardyce *et al.* 2005; Kirkbride *et al.* 2007), including income inequality (Kirkbride *et al.* 2012), have elevated incidence rates of schizophrenia; this risk may be attenuated for individuals living in communities where they can draw upon others for social support (Veling *et al.* 2008; Kirkbride *et al.* 2012). In the present study, the most parsimonious interpretation of our findings suggests that people experiencing their first episode of psychosis in more densely populated urban environments exhibited more positive and depressive psychotic symptoms and less disorganization. Our data are consistent with the possibility that aspects of the environment can alter the syndromal presentation of FEP, particularly with regard to positive psychotic phenomenology.

## Supplementary Material

Supplementary Material Supplementary information supplied by authors.

## Supplementary material

For supplementary material accompanying this paper visit http://dx.doi.org/10.1017/S0033291713003188.
