# Supplementary material for: The effect of the environment on symptom dimensions in the first episode of psychosis: a multilevel study
Source: Psychol Med. 2014 Jan 20;44(11):2419–30. doi: 10.1017/S0033291713003188 (PMC4070408; doi:10.1017/S0033291713003188)
Supplement: Supplementary Material — Supplementary information supplied by authors. [file S0033291713003188sup001.pdf]

ST1: Factor loadings of 28 IGC items for 484 people with FEP, following principal axis factor analysis with Varimax rotation

Dimensions	Factors[a]				
	1 (Manic symptoms)	2 (Reality distortion)	3 (Negative symptoms)	4 (Depressive symptoms)	5 (Disorgan-ization)
**1. Manic symptoms**					
Heightened subjective functioning	**0.86**	-0.08	-0.08	-0.06	-0.03
Rapid subjective tempo	**0.83**	-0.04	-0.06	-0.03	0.01
Expansive mood	**0.82**	-0.07	-0.07	-0.06	-0.04
Expansive delusions & hallucinations	**0.63**	0.13	-0.07	-0.07	0.07
Overactivity	**0.62**	-0.01	0.01	0.08	0.24
Socially embarrassing behavior	**0.40**	0.16	0.11	-0.12	0.22
**2. Reality distortion**					
Delusions of reference	0.11	**0.46**	-0.0009	0.21	0.09
Delusions of persecution	-0.08	**0.43**	-0.07	0.03	0.18
Delusions of control	-0.06	**0.37**	0.0002	-0.03	0.14
Bizarre delusions & interpretations	-0.09	**0.41**	-0.04	-0.15	0.23
Miscellaneous delusions	0.08	**0.38**	-0.08	0.08	0.07
Non-specific auditory hallucinations	-0.09	**0.55**	0.10	0.06	-0.33
Non-specific visual hallucinations[b]	0.003	0.24	0.03	0.03	-0.08
Non-affective auditory hallucinations	-0.08	**0.61**	0.11	-0.14	-0.30
Altered perception	0.12	**0.31**	-0.01	-0.06	0.03
Non-specific psychotic experiences	0.01	**0.34**	-0.02	0.01	-0.01
Experience of disordered form of thoughts	0.05	**0.48**	0.03	-0.08	0.12
Depersonalization & derealization[b]	-0.01	0.24	0.04	0.04	-0.004
**3. Negative symptoms**					
Flat & incongruous affect	-0.12	0.02	**0.65**	0.01	0.12
Poverty of speech	-0.07	-0.11	**0.69**	-0.007	-0.04
Non-verbal communication	0.09	-0.03	**0.73**	0.17	-0.04
Self-neglect	-0.01	0.09	**0.37**	0.02	0.19
Motor retardation	-0.09	0.01	**0.61**	**0.36**	-0.18
**4. Depressive symptoms**					
Special features of depressed mood	-0.06	0.04	0.13	**0.84**	-0.06
Depressed mood	-0.17	-0.06	0.04	**0.82**	-0.05
Depressive delusions & hallucinations	-0.09	0.005	0.13	**0.58**	-0.09
**5. Disordered beliefs**					
Emotional turmoil	0.16	0.12	0.23	-0.02	**0.38**
Incoherent speech	0.10	0.06	0.003	-0.12	**0.46**

[a] Factor loadings following Varimax rotation. Loadings ≥±0.30 are presented in bold. Loadings rounded to 2 decimal places, or 1 significant digit for values <±0.01

[b] IGC item did not load **>**±0.30 onto a specific factor, but strongest loadings were associated with *reality distortion*

ST2: Null & univariate multilevel models of symptom dimensions in the ÆSOP First Episode Psychosis sample

	Symptom dimension[a]				
	Reality distortion	Negative symptoms	Manic symptoms	Depressive symptoms	Disorganization
**Null models**					
Random effect					
Intra-class correlation coefficient	4.92%	0.74%	0.73%	<0.001%	3.11%
χ^2 p-value	0.03	0.35	0.34	1.00	0.05
AIC	539.51	1160.90	1611.43	939.26	-422.96
**Univariate models**					
Individual-level fixed effects	**EES (95%CI)**	**EES (95%CI)**	**EES (95%CI)**	**EES (95%CI)**	**EES (95%CI)**
Age (years)	-0.0001 (-0.004, 0.003)	-0.004 (-0.01, 0.003)	-0.01 (-0.02, 0.002)	0.004 (-0.001, 0.01)	0.0007 (-0.0006, 0.002)
Sex (men vs. women)	0.02 (-0.05, 0.10)	0.19 (0.04, 0.34)[b]	-0.24 (-0.49, 0.002)	-0.16 (-0.27, -0.04)[b]	0.02 (-0.005, 0.05)
White British	1	1	1	1	1
White, other ethnicities	0.19 (0.03, 0.34)[b]	0.05 (-0.25, 0.35)	-0.23 (-0.72, 0.25)	0.04 (-0.19, 0.28)	-0.01 (-0.07, 0.04)
Black Caribbean	0.13 (0.03, 0.22)[b]	-0.13 (-0.31, 0.06)	-0.05 (-0.34, 0.25)	-0.07 (-0.21, 0.08)	-0.02 (-0.06, 0.01)
Black African	0.21 (0.09, 0.33)[b]	0.02 (-0.22, 0.25)	0.28 (-0.10, 0.66)	-0.01 (-0.20, 0.18)	-0.05 (-0.09, -0.003)[b]
Indian subcontinent	-0.12 (-0.35, 0.10)	-0.28 (-0.72, 0.17)	-0.21 (-0.93, 0.52)	0.08 (-0.27, 0.44)	-0.01 (-0.19, 0.13)
Mixed, white & black Caribbean	-0.18 (-0.44, 0.09)	0.52 (-0.002, 1.04)	0.33 (-0.51,1.18)	-0.002 (-0.42, 0.41)	0.03 (-0.07, 0.12)
Other ethnicities	0.09 (-0.08, 0.26)	0.05 (-0.28, 0.38)	0.19 (-0.34, 0.73)	0.12 (-0.14, 0.38)	-0.03 (-0.10, 0.03)
Area-level fixed effects					
SE London vs. Nottinghamshire	0.22 (0.14, 0.30)[b]	-0.13 (-0.29, 0.02)	0.02 (-0.24, 0.29)	0.13 (0.002, 0.25)[b]	-0.06 (-0.08, -0.03)[b]
Population density[c]	0.10 (0.06, 0.13)[b]	-0.02 (-0.10, 0.05)	0.04 (-0.09, 0.16)	0.05 (-0.01, 0.11)	-0.03 (-0.04, -0.01)[b]
Deprivation (IMD)[c]	0.04 (-0.01, 0.10)	-0.02 (-0.11, 0.08)	-0.10 (-0.24, 0.06)	-0.07 (-0.15, -0.001)[b]	-0.01 (-0.03, 0.004)
Inequality (IMD)[c]	-0.06 (-0.12, -0.01)[b]	-0.003 (-0.10, 0.10)	0.04 (-0.12, 0.19)	0.009 (-0.07, 0.09)	0.02 (0.0006, 0.04)[b]
Own-group ethnic density[c]	-0.07 (-0.11, -0.02)[b]	0.03 (-0.05, 0.11)	0.01 (-0.11, 0.14)	-0.004 (-0.07, 0.06)	0.02 (0.003, 0.03)[b]
Local election voter turnout[c]	-0.08 (-0.14, -0.03)[b]	0.07 (-0.03, 0.17)	0.04 (-0.13, 0.20)	0.02 (-0.06, 0.10)	0.03 (0.008, 0.05)[b]

AIC: Akaike Information Criterion; 95%CI: 95% confidence interval; EES: estimated effect size on transformed scale; IMD: Index of Multiple Deprivation

[a] Figures rounded to 2 decimal places, or where between -0.01 and 0.01 rounded to 1 significant digit.

[b] Significant at p<0.05

[c] Z-standardized effect sizes correspond to change in symptom dimension associated with a 1 standard deviation change in the neighborhood variable, except for the centre-variable which reports change in effect size in Southeast London compared with Nottinghamshire

ST3: Multivariate adjusted area-level effects in selected symptom dimensions by broad ethnic subgroup

	Symptom dimension[a]		
	Reality distortion EES (95% CI)	Depressive symptoms EES (95% CI)	Disorganization EES (95% CI)
***Area-level fixed effect* (Southeast London vs. Nottinghamshire)**			
White British group	0.13 (0.003, 0.26)[b]	0.24 (0.06, 0.473[b]	-0.05 (-0.10, -0.01)[b]
Black and Minority Ethnic group	0.21 (0.07, 0.34)[b]	0.15 (-0.07, 0.37)	-0.09 (-0.14, -0.03)[b]

EES: estimated effect size; OR: odds ratio; 95% CI: 95% confidence interval
[a] Effect sizes adjusted for all variables included in final models presented in Table 3.
[b] $p<0.05$
